# Specialty grand challenge in plant biophysics and modeling

**DOI:** 10.3389/fpls.2022.991526

**Published:** 2022-09-02

**Authors:** Ingo Dreyer

**Affiliations:** Centro de Bioinformática, Simulación y Modelado (CBSM), Facultad de Ingeniería, Universidad de Talca, Talca, Chile

**Keywords:** modeling, plant biophysics, computational cell biology, mathematical model, structural model

## Introduction

Plant biophysics and modeling is, to be honest, still an exotic field in Plant Science. By its very definition, it is highly interdisciplinary and involves, besides different aspects of plant biology, mathematics, physics, and chemistry. Nevertheless, it holds great untapped potential that could catapult plant science forward in much the same way as the molecular revolution has in recent decades. The enormous potential of describing complex systems in a logically consistent and explicit manner may be illustrated by the growth of the discipline of physics in the eighteenth to twentieth centuries that also strongly influenced mathematics. On that mathematical basis, nowadays, the increasing availability of computing power has opened up many more possibilities to delve into complex systems that seemed unattainable only some time ago. We should therefore be aware that for modeling in plant science, computational power is not necessarily the limiting resource any longer. However, there are other obstacles that need to be tackled (see the Challenges below).

Plant biophysics and modeling is diverse, ranging from the molecular to the organismic level. It covers the areas of molecular and cell biophysics, physiology, bionics, computational cell biology, structural protein analyses, synthetic biology, quantitative plant development, and biomechanics. In particular the computational approaches are new and just beginning to unleash their potential impact. At the molecular level, they open up new possibilities in structural modeling, and at the cellular and organismal levels, they contribute fundamentally to mechanistic mathematical modeling.

### Structural modeling

Knowledge of protein structures is key to understanding biochemical processes in cells. Solving the first protein structure by X-ray crystallography in the 1950s was an initial breakthrough to explore the molecular level. Since then, this technique has been refined and was complemented by nuclear magnetic resonance (NMR) spectroscopy in the 1970s/1980s and recently by cryogenic electron microscopy (cryo-EM). In addition structural information is also obtained from modeling [template-based, ab initio, Monte Carlo (MC), and Molecular Dynamics (MD)] (Rasheed et al., 2020). Meanwhile the archive of the Protein Data Bank (PDB) contains about 200,000 entries as 3D structures of biomolecules at the atomic level with an annual increase of more than 10,000 structures (https://www.rcsb.org/stats/growth/growth-released-structures). Although this development is impressive, the major drawback is that less than 9% of PDB entries are from plant proteins, most likely due to the lower resources available compared to medical research. Nevertheless, with AlphaFold (https://www.deepmind.com/research/highlighted-research/alphafold), the next stage in protein research is just being ignited that may overcome this obstacle. This program is a deep learning system developed by Alphabet's/Google's DeepMind which performs predictions of protein structure. Although we should handle the predicted structures with due caution and skepticism, we can expect the number of available structures to literally explode soon. With the expected increasing accessibility of plant protein structures, efforts can be intensified to gain detailed knowledge of molecular processes in plants. And here, computational simulations such as docking or Molecular Dynamics simulations will play an increasingly crucial role in the near future (Navarro-Retamal et al., 2016a,b, 2018, 2021; Khan et al., 2019; Schott-Verdugo et al., 2019; Rasheed et al., 2020; Lehmann et al., 2021; Moe-Lange et al., 2021; Peña-Varas et al., 2022).

### Mechanistic mathematical modeling

The fundamental basis for the correct description of complex biological systems, such as we find in plants, is the mathematical representation of the individual entities. Only in this way is it possible to use computers to simulate the dynamics of the systems in mechanistic mathematical models (Dale et al., 2021). Such models are often designed to mimic the physiological reality in order to support experimental data (see for instance, Chen et al., 2012; Hills et al., 2012; Blatt et al., 2014; Morris, 2018; Holzheu and Kummer, 2019; Iosip et al., 2020; Huang et al., 2021; Jezek et al., 2021). However, they are not limited to this. Mechanistic mathematical models and subsequent computer simulations enable rigorous investigation of our hypotheses about phenomena even without exhaustive data. Such solidly based thought experiments allow to test also conditions that are hard to achieve in conventional wet-laboratory experiments and by doing so they have an inestimable value in gaining new insights. For instance, minimal models for the nutrient exchange between plants and fungi suggest that in symbiosis the “cooperation” between plant and fungus is the result of competition between both for the same resources in the tiny space of their direct contact zone (Schott et al., 2016; Dreyer et al., 2019; Nizam et al., 2019). Interestingly, the models further indicate that the boundary between symbiosis and parasitism depends on the strength of the partners, as both situations rely on the same mechanisms for nutrient exchange (Wittek et al., 2017). But, even seemingly unalterably firmly established dogmas, such as that of high- and low-affinity transport in plants, can be tested in simulations and revised, if necessary (Dreyer and Michard, 2020). This ultimately opens the door to new, alternative concepts that are compatible with basic thermodynamic principles and offer a different perspective for our physiological understanding (see for instance, Wegner and Shabala, 2020; Dreyer, 2021; Wegner et al., 2021; Dreyer et al., 2022).

Mechanistic mathematical modeling has proven to be able to provide fundamental insights into the dynamics of plant systems. More importantly, it has preserved us already from too many dead-end strategies to be tested in the wet-lab and in field trials. They therefore contribute to the efficient use of available resources for research. Nevertheless, despite the huge potential of mechanistic mathematical models and computational cell biology simulations, there are significant obstacles to implementing these approaches (Dale et al., 2021). Some of the biggest challenges are outlined in the following.

## Challenge 1: Interdisciplinary education

For far too long time, we divided natural sciences into more and more disciplines and subdisciplines that with the time lost partially the links between them. With the growing level of knowledge, this was of course inevitable. Specialization is necessary because no one can have expert knowledge in all areas, but it also carries the danger that we no longer see the big picture due to all the information about the small details. In this area of tension, the question arises at what point specialization makes sense. Unfortunately, we have adopted the advanced subdivision of science deep into our secondary educational system. For instance, in the author's school days, biology was to a large extent a purely descriptive subject, while physics was dominated by mathematics. In terms of teaching content, a connection between biology and physics was beyond any imagination, although there were certainly interconnections between these disciplines at universities and research centers. But by the time these integrating approaches became visible in training, the dividing walls were usually already firmly anchored in the minds and then had to be painstakingly torn down again. With the modern buzzwords “multi- and interdisciplinarity,” public perception is now changing and the need to soften the strict separation between disciplines of natural sciences in education is emerging. Scientific interdisciplinary content for Young Minds (e.g., https://kids.frontiersin.org/) is therefore invaluable and we should always ask ourselves in our publications: How can we present our research in a way that it is easy for others (non-specialists) to grasp? (see also Challenge 4).

## Challenge 2: Interdisciplinary teambuilding

Even with a robust interdisciplinary training there is still the need to gather different experts to understand complex systems. The key obstacle in teambuilding is often to find a proper basis of mutual understanding of the limitations of the different approaches. For example, a modeler might want a basis of experimental data that a wet-lab experimenter would consider realistically unattainable. The other way around, it could be that wet-lab scientists expect a model with a richness of detail, which a modeler cannot provide due to various difficulties. Without reciprocal tolerance and understanding, a started collaboration can end faster than expected in a premature stage. In fact, the greater the gap between the backgrounds of different scientists, the more challenging the learning process on all sides. But the author can assure from his own experience: The effort is worth it, and with every successfully completed project, the mental gap between the (sub-)disciplines narrows. Patience really pays off.

## Challenge 3: Balance realism with parsimony

The mechanistic modeling of complex systems always moves in a field of tension between realism and abstracting simplification. Unfortunately, it sometimes happens that people (often without modeling experience) reject the results of a modeling approach on the grounds that the model is an oversimplification of reality. In this conflict, it is worth asking what the actual goal of the model should be: Should the underlying mechanism be worked out or should the experimental data be modeled as accurately as possible? Often, the first goal comes at the expense of the second. In order to highlight general features of a complex system, some details have to be suppressed, otherwise one can no longer see the forest for the trees. A feasible guiding principle in this challenge might be: Make a mechanistic model as simple as possible but as complex as necessary.

## Challenge 4: Didactic presentation

In order to effectively reach a wide range of colleagues and to promote their field, researchers need to take responsibility to communicate their discoveries in a manner that is accessible to a wide spectrum of scientists. Mechanistic mathematical models might be seen by many readers as cryptic black boxes if they are not didactically clearly presented and discussed. When modeling, we should always be aware that there is the danger to hide simple relationships behind a complex mathematical framework. In this context, adherence to the guiding principle “as simple as possible, but as complex as necessary” is an essential first step. But also the way of presentation is of enormous importance. The adage “a picture is worth a thousand words” could be mentioned here, because keeping to it helps in nailing down the storyboard of an article. When a story is already tangible through the figures, it is much easier to write. In this context, online journals, like Frontiers in Plant Science, offer opportunities that should be used: (1) The space limit is less rigid than in printed journals, which allows also more figures, and (2) figures can be generally in color without extra cost. I therefore encourage us all to be as creative in the didactic presentation of our results as we are in the creation of models.

## Author contributions

ID conceived the project and wrote the manuscript.

## Funding

This work was supported in part by the Agencia Nacional de Investigación y Desarrollo (ANID) of Chile (Fondecyt Regular No. 1220504).

## Conflict of interest

The author declares that the research was conducted in the absence of any commercial or financial relationships that could be construed as a potential conflict of interest.

## Publisher's note

All claims expressed in this article are solely those of the authors and do not necessarily represent those of their affiliated organizations, or those of the publisher, the editors and the reviewers. Any product that may be evaluated in this article, or claim that may be made by its manufacturer, is not guaranteed or endorsed by the publisher.
